# Human Liver Transplantation

**Published:** 1970-07

**Authors:** M. G. Johnson

**Affiliations:** Research Assistant in the Department of Surgery, University of Bristol


### Human Liver Transplantation

**M. G. Johnson, M.B., B.S., F.R.C.S.**

Research Assistant in the Department of Surgery, University of Bristol.

When Welch (1955) reported his first experimental liver transplants in dogs, he wrote "it is interesting to speculate about the possibilites of transplanting the human liver in part or as a whole. The two problems of donation and technique of operation seem by themselves to be insurmountable obstacles with our present knowledge. Nevertheless, if some kind of assistance from a donor liver were available to patients in liver failure it would serve a most useful purpose."

The technique he described was that of auxiliary heterotopic transplantation, leaving the recipient liver in situ and using the homograft as an extra liver. Subsequently, Moore et al., (1959), and Starzl et al., (1960) published their experimental work on orthotopic transplantation, removing the animal's own liver and replacing it by the donor organ. They found that in the animals surviving for more than a few days rejection of the homograft occurred, and in later publications, Starzl et al., (1961), (1964), described the histological appearances associated with this.

It was thought that liver homotransplantation might have clinical application in certain cases of primary hepatoma or carcinoma of the biliary tract, cirrhotic patients with failing liver function and infants with congenital biliary atresia. The number of such potential recipients in England and Wales has been estimated at approximately 600 per year, Terblanche and Riddell (1967). Human liver transplantation, however, was not possible until methods of suppressing the rejection reaction became available. The drugs azathioprine and prednisone were first used in renal transplantation and shown to be of value in this respect in the latter part of 1962, and in March 1963 Starzl, in Denver, performed the first human liver transplant, Starzl (1963). In the following six years a total of 79 liver transplants were carried out in various centres throughout the world. The problems of obtaining donor organs and technique of operation have indeed proved to be difficult, but the increasing knowledge gained from laboratory and clinical work in all aspects of transplantion has made progress possible, and several patients have survived for over a year.

Progress in renal transplantation has been more rapid. By 1969 over 2,000 cases had been reported, of which just under half were cadaveric organ transplants. There are several reason why liver transplantation has not advanced so rapidly, the chief one being that the liver is much more susceptible to ischaemia at body temperature than the kidney. Whereas the liver suffers severe irreversible damage after 15 to 20 minutes following the death of the donor, the kidney is able to withstand up to 2 hours of warm ischaemia, White et al. (1968). The difficulties of obtaining viable organs and preserving function are therefore much increased in liver transplantation. In addition, the technical problems of the operation are considerably greater, and lastly, there is no method comparable to renal dialysis to support failing liver function in patients before and after transplantation.

An outline of the results of human liver transplantation will be given and then the problems that have been encountered will be discussed.

### AUXILIARY HETEROTOPIC TRANSPLANTATION

The theoretical advantages of this procedure were considered to be that it would avoid the time consuming and frequently difficult operation of hepatectomy in the recipient, and that any residual function possessed by the recipient liver might be of assistance in the early post-operative period if homograft function was impaired. It has been attempted in 24 patients with non-malignant liver disease, the majority with congenital biliary atresia and a few with cirrhosis, but the results have been very discouraging. The longest surviving patient lived 34 days, and only 3 patients lived longer than 3 weeks. The theoretical advantages were outweighed by technical difficulties in achieving a satisfactory blood supply to the homograft, and the effects of abdominal overcrowding. Further research into this procedure is being carried out, as it would probably have the widest clinical application if the problems mentioned could be solved.

### ORTHOTOPIC TRANSPLANTATION

Fifty-five orthotopic transplants have been performed. The majority of the patients had malignant hepatoma or congenital biliary atresia. The early cases were disappointing in that none of the nine patients lived beyond 23 days. Subsequently, however, improvements in the quality of the homografts used, better methods of liver preservation and control of immunosuppression led to more encouraging results.

The first long term survival was achieved in July, 1967, Starzl et al., (1968a). Since that time 46 patients have been treated by transplantation. Twenty-two survived for one month, 7 for 6 months and 5 for more than a year.

The longest and most fully documented series of orthotopic liver transplants have been from Denver, Starzl et al., (1963), (1968a), (1968b), (1969), and from the combined team at Addenbrooke's Hospital, Cambridge, and King's College Hospital, London, Calne and Williams (1968), Williams et al., (1969), Calne (1969).

The Denver series consisted of 25 patients. Twelve were infants with congenital biliary atresia, and 7 of them survived for more than 2 months. Four of these however died within 6 months from the complication of septic hepatic infarction, which will be discussed later, and three lived for over a year. Eleven patients received transplants for malignant hepatoma and there were 4 survivors for periods of $4\frac{1}{2}$ to $14\frac{1}{2}$ months. All 4 patients, however, developed recurrence of their primary disease. Two cirrhotic patients both died soon after transplantation.

The overall survival in this series was:

| | | | | | | |
|---|---|---|---|---|---|---|
| Number of months after transplant: | 1 | 2 | 3 | 6 | 9 | 12 |
| Number of patients remaining alive: | 13 | 11 | 10 | 7 | 6 | 5 |

In July 1969 there were 3 long term survivors still alive after transplantation for biliary atresia at 12, 14, and $17\frac{1}{2}$ months. However, two of these patients were jaundiced due to chronic rejection of the homograft.

Calne and Williams reported 12 orthotopic transplants. Seven of these patients had malignant disease of the liver or biliary tract. There were 3 patients with cirrhosis, one with subacute hepatic necrosis and one with congenital biliary atresia. Eight patients survived the immediate postoperative period, and five were able to leave hospital. Six patients subsequently died at 6 days and 3, 7, 10, 11 and 19 weeks post-operatively. 1 from rejection, 2 from pneumonia and 2 from biliary tract complications. Two patients are now alive and well 10 and 11 months after transplantation for cirrhosis and hepatoma, Williams (1970, Personal communication).

Many factors have affected the outcome of these procedures, but the main ones have been the quality of homograft used, technical difficulties at the time of operation, the rejection reaction and the complications of immunosuppressive drugs, and lastly, recurrence of malignant disease.

### THE QUALITY OF THE HOMOGRAFT

Liver function becomes irreversibly damaged if the organ remains at body temperature without a recirculation for more than 15 to 20 minutes. If the organ is rapidly cooled within this time, satisfactory function can be preserved provided it it revascularised in the recipient within 2 or 3 hours. The liver may have been subjected to a period of warm ischaemia before the death of the donor, depending on the mode of death and the duration of any preceding hypotension. One of the main causes of failure following the early human liver transplants was the use of homografts that had undergone ischaemic damage before death of the donor, during prolonged agonal hypotensive periods. The recipients, in these cases, developed liver failure postoperatively. The ensuing bleeding diathesis caused the death of one patient, and made haemostasis extremely difficult to achieve in the others. In later cases, when donor selection was more discriminating and methods of liver preservation had been improved, the homograft function was satisfactory.

The most suitable donors are patients for whom resuscitation has been attempted by mechanical ventilation and other means of support, but in whom it is eventually decided to abandon efforts at resuscitation because of irreversible brain damage, irrespective of any transplant considerations. In such circumstances, there is sufficient time available to make the many arrangements necessary before transplantation. Following cessation of heart beat and spontaneous respiration in such cases, the period of warm ischaemia is kept to a minimum, either by external cardiac compression followed by rapid infusion of a cold balanced electrolyte solution via the portal vein, or the use of extracorporeal hypothermic perfusion of the cadaver. Preservation of function after the initial cooling of the liver, was achieved in Calne's cases by infusing a physiological conservation medium into the liver, and keeping the organ at 4 degrees Centigrade. This method described by Schalm (1968) has been shown experimentally to maintain liver function for 3 to 4 hours, but in human transplants, Calne (1969), the total period of " cold ischaemia " has not exceeded 2 to 3 hours. A more complicated, but more efficient method has been used in the majority of the last 18 transplants in Denver. These homografts were stored for periods up to $3\frac{1}{2}$ hours in isolation, but a technique combining hypothermic perfusion and hyperbaric oxygenation, Brettschneider et al., (1968). The maximum interval from donor death to revascularisation of the homograft was $7\frac{1}{2}$ hours, and the recipient lived for over a year.

### TECHNICAL COMPLICATIONS

The most serious hazards arose in connection with the vascular anastomoses, and the biliary drainage of the homograft. Vascular complications were particularly common in cases of congenital biliary atresia, due to the extremely small calibre of the hepatic arteries, and the increased incidence of anatomical anomalies of the hilar vessels. Four such infants in the Denver series died soon after operation, 3 from hepatic artery occlusion and 1 from portal vein thrombosis. The development of fatal septic hepatic infarction in four more infants was largely due to kinking and thrombosis of the right branch of the hepatic artery produced by rotation of the liver postoperatively, Starzl (1968b). In subsequent cases, this complication was prevented by anchoring the liver to the diaphragm. Only one adult in the Cambridge series developed hepatic artery thrombosis.

The profound changes in blood coagulation associated with orthotopic liver transplantation have not only led to difficulty in achieving haemostasis in some cases, especially when a poorly functioning homograft was transplanted, but may also have contributed to the incidence of vascular thrombosis in other cases. Studies by von Kaulla (1966), Blecher (1968), and Pechet (1969), suggested that increased intravascular coagulation occurred associated with secondary fibrinolysis. Flute (1969) also found evidence of intravascular coagulation following human liver transplantation, and emphasised the possible importance of this in the production of vascular occusion, especially following the use of antifibrinolytic drugs.

The presence of anatomical anomalies of the homograft biliary tract resulted in the death of 2 of Starzl's patients from iatrogenically produced biliary obstruction. The technique of biliary drainage used has varied. Theoretically, preservation of the sphincter of Oddi is preferable to prevent ascending cholangitis, but in three of five cases where end to end anastomosis of the common bile duct was performed, with T tube splintage, biliary fistulae developed, Calne (1969). Calne found anastomosis of the homograft gallbladder to the recipient common bile duct gave better results. This method, however, is not applicable to cases of biliary atresia. Starzl used cholecystoduodenostomy, and the incidence of cholangitis was very low.

### REJECTION AND IMMUNOSUPPRESSION

It appeared from the early experimental work on liver homotransplantation in dogs that the rejection process was marked in the majority of cases. It was later found that in the pig this process was very mild, and that long term survival was possible without the use of immunosuppressive drugs, Peacock and Terblanche (1967). It was suggested that the presence of hepatic vein sphincters in the dog, causing outflow block and ischaemic damage, may have accentuated the rejection process seen in this animal. Neither the pig liver nor the human liver possess these sphincters, and it was therefore felt that rejection in human liver homografts might also prove to be relatively mild.

Immunosuppressive drugs have been used in all except one case following human liver transplantation. In this instance they were withheld for fear of flaring up a viral hepatitis. The patient developed acute homograft rejection 4 days post-operatively, and died 2 days later, Williams et al., (1969). Homograft rejection was seen in the majority of the other patients, but was modified by drug therapy. In some cases the onset of this process was early, within a month of operation, but in others it was delayed for 2 to 6 months.

The early rejection episodes in the Cambridge series were controlled by a temporary increase in the dose of prednisone. Starzl described three types of early rejection. The mildest form was not accompanied by jaundice. In others the onset was so abrupt and the symptoms and signs were so marked that they were termed rejection crises. It was found, however, that both these types of rejection reversed spontaneously, without increasing the dose of immunosuppressive drugs, and that the severity of the rejection crises did not preclude long term survival. The third variety was indolent in nature, and proved to be difficult or impossible to reverse. One patient received a second transplant because of progressive indolent rejection.

Chronic homograft rejection resembled the early indolent type except that it was later in onset. All the patients surviving for prolonged periods in Starzl's series were affected, and in only one case was the process reversible. Although liver function slowly deteriorated, the chronically rejecting homograft supported life for many months, and 3 patients survived for over a year. The development of indolent or chronic rejection appeared to be related to difficulty in maintaining immunosuppressive therapy in these patients. They all received antilymphocyte globulin (A.L.G.) in addition to prednisone and azathioprine. The advantage of A.L.G. was that it allowed a smaller dose of azathioprine to be used, and hence reduced the risk of leukopenia. A serious disadvantage, however, was the development of anaphylactic or local reaction to the foreign protein, which made it impossible to continue the injection in many cases. Indolent or chronic rejection frequently occurred when the drug was stopped. The most satisfactory case in the whole series was the only one in which it was possible to continue long term treatment with A.L.G. It therefore seemed from Starzl's experience that A.L.G. was valuable in controlling the rejection process, but that a state of dependence developed.

By contrast, only 3 patients in the Cambridge series were given A.L.G. in addition to the other two drugs, for short periods up to 3 weeks, and no signficant advantage was found. Neither of the 2 patients, who are now alive and well 10 and 11 months after transplantation, received it. Chronic rejection was seen in only one case in this series, and could not be reversed despite massive doses of drugs. In this instance, the donor-recipient tissue match was poor.

No association could be found, in the Denver series, between the degree of donor—recipient histo-compatibility as judged by tissue typing, and the patterns of rejection observed, Terasaki (1969). The variations in immunosuppressive therapy played a large part in determining the outcome even when the best tissue match was obtained. A significant correlation has, however, been found between histo-compatibility matching and survival following cadaveric renal transplantation, van Rood (1969), and it is likely that this will also apply in the case of liver transplantation.

The major disadvantage of non-specific immunosuppressive therapy is an increased susceptibility of the patients to infection, and this has been an important cause of morbidity and mortality following liver transplantation. The patients were very prone to chest infections and localised sepsis tended to spread rapidly. The responsibile organisims were frequently Gram negative bacilli and fungi, and the infection often followed broad spectrum antibiotic therapy. Starzl found that when the quality of the homograft transplanted was improved, the incidence of infective complications was reduced. Intermittent bacteraemia was found in some patients at various times after operation in both the Cambridge and Denver series. Williams et al., (1969) found the organisms were frequently the same as those isolated in the bile, but there was no evidence of cholangitis or liver infarction, and antibiotics were not given. Starzl (1969a) felt from his experience, that patients were particularly prone to infection after liver transplantation, and postulated that a deficiency of the " bacterial filtering " functions of the liver allowed access of gastrointestinal organisms to the circulation.

### RECURRENCE OF DISEASE AFTER LIVER TRANSPLANTATION

All 4 patients in Starzl's series who survived for prolonged periods after transplantation for malignant hepatoma, developed recurrence of their primary disease. Pulmonary metastases were diagnosed in 3 cases on chest X-rays between 4 and 13 weeks post-operatively, and the rate of tumour growth appeared to be very rapid. In all cases recurrence of the tumour involved the homografts, and in one case replaced virtually the whole of it. These results were very disappointing, although not completely unexpected in view of the highly malignant nature of these tumours, the prognosis in most cases being less than 6 months from the time of diagnosis, Lawrence et al., (1966). The original tumours were very large, and microscopic spread of malignant cells must have occurred pre-operatively. Starzl (1969b) commented that the rate of recurrence may have been accelerated by the use of immunosuppressive drugs. However, one of Calne's patients remains alive and well 11 months after transplantation for hepatoma, and liver biopsy now shows essentially normal histology, Williams (1970, Personal communication).

### CONCLUSIONS

The high morbidity and mortality associated with liver transplantation is not surprising in view of the poor state of health of many of the recipients pre-operatively, and the numerous problems that have been encountered. Technical calamities and the use of unsuitable donor organs accounted for over half of the mortality in the Denver series. Nevertheless, in a number of patients, symptoms have been relieved and 5 patients have survived for a year or more. Two others are alive and leading reasonably normal lives 10 and 11 months after operation.

Each of the 3 groups of potential recipients present particular problems. In congenital biliary atresia these are the technical difficulties with the vascular anastomoses and the shortage of suitably small sized donor organs. Whether patients with malignant hepatoma, where the disease is still localised to the liver, but beyond the scope of conventional surgery, should still be considered for transplantation in view of the results so far, is debatable. It may be that if patients with less advanced tumours than the ones in Starzl's series were treated, that the results would be better. It appears to be essential to remove the tumour completely if there is to be any hope of avoiding recurrence. Carcinoma of the extrahepatic ducts is a more slowly growing tumour, and although it does not produce symptoms till late, spread beyond the liver and regional lymph nodes is rare. The less malignant nature of this tumour might make it more suitable for transplant surgery than hepatoma.

Cirrhotic patients with failing liver function form the largest group of potential recipients in this country. Terblanche and Riddell (1967) estimated the number to be about 300 per year in England and Wales. The prognosis of the "better risk" cirrhotic patient is difficult to predict with absolute certainty, and there is therefore reluctance to undertake transplantation until the patient is in a terminal state. At present, there is no method of maintaining failing liver function until a suitable donor organ becomes available. If the problems associated with auxiliary liver transplantation can be overcome, this procedure would be particularly applicable to the cirrhotic patient with a small shrunken liver and long standing abdominal distension due to ascites.

Further advances in methods of immunosuppression and organ storage are required before the full potential value of liver transplantation can be realised. The present methods of immunosuppression, although useful, are far from ideal because of their many side effects. A less toxic form of antilymphocyte globulin is required before its full value can be assessed. Some objective way of measuring the degree of rejection, and the effect of immunosuppressive drugs on it, would be extremely useful. The ultimate aim in organ transplantation is the development of methods of inducing donor specific tolerance in the recipient.

The development of a transportable storage unit, capable of preserving liver function longer than 8 to 12 hours, would greatly facilitate the organisation of liver transplantation. This would allow a donor organ to be used for the most suitable recipient, as judged by tissue typing, and enable a planned procedure rather than " an emergency " operation to be carried out at the centre best equipped for transplantation. In these circumstances, it would obviously be valuable to have some means of assessing the viability of the homograft before transplanting it. It will be some while before all these objectives are realised.

It has been shown in the centres with the most experience, that even with the techniques at present available, prolonged survival can be achieved in about a fifth of the patients. It would therefore seem reasonable to offer liver transplantation to selected patients with liver disease, having a prognosis of less than six months. The shortage of donor organs however, is a major problem as in all forms of cadaveric organ transplantation, and will remain so until satisfactory methods of long term storage have been developed.

### REFERENCES

**Blecher, T. E., Terblanche, J., and Peacock, J. H.** (1968). Annals of Surgery, **96,** 331.

**Bretischneider, L. et al.** (1968). Surgery. Gynecology and Obstetrics, **126,** 263.

**Calne, R. Y.** (1969). British Journal of Surgery, **56,** 10. 729.

**Calne, R. Y., and Williams, R.** (1968). British Medical Journal, **4,** 535.

**Flute, P. T. et al.** (1969). British Medical Journal, **3,** 20.

**Lawrence, G. H. et al.** (1966). American Journal of Surgery, **112,** 200.

**Moore, F. D. et al.** (1959). Transplantation Bulletin **6,** 103.

**Peacock, J. H., and Terblanche, J.** (1967). Orthotopic homotransplantation of the liver in the pig. In The Liver, Colston Papers. London, Butterworths p. 333.

**Pechet, L. et al.** (1969). Journal of Laboratory and Clinical Medicine, **73,** 91.

**Schalm, S. W.** (1968). A Simple and Clinically Applicable Method for the Preservation of a Liver Homograft. Thesis. University of Leiden.

**Starzl, T. E. et al.** (1960). Surgery, Gynecology and Obstetrics, **111,** 733.

**Starzl, T. E. et al.** (1961). Surgery, Gynecology and Obstetrics, **112,** 135.

**Starzl, T. E. et al.** (1963). Surgery, Gynecology and Obstetrics, **117,** 659.

**Starzl, T. E. et al.** (1964). Annals of Surgery, **160,** 411.

**Starzl, T. E. et al.** (1968a). Surgery, **63,** 549.

**Starzl, T. E. et al.** (1968b). Annals of Surgery, **168,** 392.

**Starzl, T. E.** (1969). Experience in Hepatic Transplantation, ed. Starzl, T. E. and Putnam, C. W.—W. B. Saunders.

**Starzl, T. E.** (1969a). ibid p. 346.

**Starzl, T. E.** (1969b). ibid p. 369.

**Terasaki, P. I.** (1969). ibid p. 30.

**Terblanche, J., and Riddell, A. G.** (1967). The Strategy of Liver Transplantation. In The Liver, Colston Papers, London, Butterworths, p. 321.

**van Rood, J. J.** (1969). Lancet, **1,** 1142.

**von Kaulla, K. N. et al.** (1966). Archives of Surgery, **92,** 71.

**Welch, C. S.** (1955). Transplantation Bulletin, **2,** 54.

**White, H. J. O., Evans, D. B., and Calne, R. Y.** (1968). British Medical Journal, **4,** 739.

**Williams, R. et al.** (1969). British Medical Journal, **3,** 12.

